# Effects of *Lactobacillus plantarum* CQPC01‐fermented soybean milk on activated carbon‐induced constipation through its antioxidant activity in mice

**DOI:** 10.1002/fsn3.1048

**Published:** 2019-05-01

**Authors:** Bihui Liu, Xiao Xiao, Xianrong Zhou, Jie Zhou, Lingxia Lan, Xingyao Long, Yanni Pan, Muying Du, Xin Zhao

**Affiliations:** ^1^ Chongqing Collaborative Innovation Center for Functional Food Chongqing University of Education Chongqing China; ^2^ Chongqing Engineering Research Center of Functional Food Chongqing University of Education Chongqing China; ^3^ Chongqing Engineering Laboratory for Research and Development of Functional Food Chongqing University of Education Chongqing China; ^4^ College of Biological and Chemical Engineering Chongqing University of Education Chongqing China; ^5^ Department of Gastroenterology Emergency Medical Center of Chongqing The Affiliated Central Hospital of Chongqing University Chongqing China; ^6^ College of Food Science Southwest University Chongqing China; ^7^ Department of Food Science and Biotechnology Cha University Seongnam Korea

**Keywords:** gene, *Lactobacillus plantarum* CQPC01, mice, pickled cabbage, soybean isoflavone

## Abstract

A newly found strain, *Lactobacillus plantarum* CQPC01 (LP‐CQPC01), was used for soybean milk fermentation, and its effects against constipation were determined. LP‐CQPC01‐FSM (LP‐CQPC01‐fermented soybean milk) was found to have six kinds of soybean isoflavones; the isoflavones of LP‐CQPC01‐FSM were more than those of *Lactobacillus bulgaricus*‐fermented soybean milk (LB‐FSM) and unfermented soybean milk (U‐FSM). Animal experiment showed that the MTL, Gas, ET, AchE, SP, VIP, and GSH levels in the constipated mice were increased; however, the SS, MPO, NO, and MDA levels in the constipated mice were reduced by soybean milk treatment. Further, LP‐CQPC01‐FSM increased the mRNA and protein expression of Cu/Zn‐SOD, Mn‐SOD, CAT, c‐Kit, SCF, and GDNF and reduced the expression of TRPV1 and NOS relative to those of the mice with untreated constipation. LP‐CQPC01 could be used as a new starter to produce high‐quality soybean milk, which might be used as a functional drink.

## 1 INTRODUCTION

There are a large number of lactic acid bacteria in pickled cabbage, and some of these strains have probiotic potential. Except for probiotics, harmful bacteria are found in the intestinal tract. Under normal circumstances, the two species are in a state of equilibrium (Garciamarengoni & Menezesalbuquerque, 2017). Probiotics in the gut are involved in digestion. Without probiotics, indigestion and digestive tract dysfunction would occur (Simrén et al., 2013). Lactic acid bacteria in the gastrointestinal by the metabolism of lactic acid can effectively inhibit the growth and reproduction of harmful bacteria, as well as maintain ecological balance in the intestines and the normal function of the intestine; however, they also increase water content in feces, moistening of the bowel, and bowel movement. Chronic diarrhea, constipation, abdominal distention, dyspepsia, and other symptoms are related to imbalance of lactic acid bacteria in the intestine (Ianiro et al., 2013). Lactic acid bacteria can not only activate macrophage phagocytosis but also play an active role in intestinal colonization. In addition, lactic acid bacteria can stimulate peritoneal macrophages, induce interferon, promote cell division, produce antibodies, promote cellular immunity, enhance the nonspecific and specific immune responses, and improve resistance to disease. Therefore, lactic acid bacteria can promote peristalsis and defecation through various ways (Pavan, Desreumaux, & Mercenier, 2003; Sierra et al., 2010). Many studies have shown that lactic acid bacteria can improve the intestinal environment through various ways and enhance intestinal vitality, thereby maintaining intestinal health and relieving constipation (Ding et al., 2016; Mohamed Mahzir, Abd Gani, Hasanah Zaidan, & Halmi, 2018; Suo et al., 2014).

A clinical study has shown that oxygen free radicals and lipid peroxidation are significantly higher in patients with constipation than those in healthy subjects (Ren, Zhang, Liu, & Cao, 2003). Oxygen free radicals and lipid peroxidation play an important role in the metabolism of the human body. The disorder of oxygen free radicals and lipid peroxidation in the body causes disorders of metabolism and immune function, as well as diseases, including constipation (Zhou & Fei, 1995). Meanwhile, the elderly have different degrees of constipation with an increase in age. The proportion of constipation in the elderly is 25%–32%; this percentage increases as age increases. These results show that the oxidative senescence of the body directly correlates with constipation (Zhang et al., 2015). Control of oxidative stress and free radicals in the body may inhibit constipation. A study showed that lactic acid bacteria cannot directly reach the oxidative stress position and reactive oxygen species; however, lactic acid bacterial cells and cell‐free extracts exhibited a similar antioxidant activity in vitro, and intake of lactic acid bacteria in the body exerted significant regulatory effects on oxidative stress in the body or cell (Baba, Najarian, Shori, Lit, & Keng, 2014).

Active substances in food are the key to their nutritional and functional functions. Including extracting effective active substances from food waste is a good way to develop functional ingredients (Jabri et al., 2017; Venturi et al., 2017). The antioxidative substances obtained by multiple extraction methods will increase the probiotics in the intestinal tract and benefit the health of the human body, including the inhibition of constipation, inflammation, and even cancer (Hochstein & Atallah, 1988; Jabri et al., 2017; Valtueña et al., 2008; Venturi et al., 2017). Soybean is a common food, which contains abundant physiological active substances, including soybean isoflavone, soybean protein, soybean phospholipid, soybean sterol, soybean saponins, soybean oligosaccharides, soybean dietary fiber, and so on (Piastowska‐Ciesielska & Gralak, 2010). Soybean isoflavone is a kind of the most important active ingredients in soybean, including daidzein, daidzin, genistin, genistein, glycitin, and glycitein. Soybean isoflavones have a clear antioxidant effect, which can also play a role in the prevention of Alzheimer's disease, cardiovascular disease, and cancer (Gutierrez‐Zepeda et al., 2005). Soybean isoflavones are flavonoid compounds, which are secondary metabolites formed during soybean growth, and constitute a kind of bioactive substance. Genistein contains 5,7,4 three phenolic hydroxyl groups, and daidzein contains 7,4 two phenolic hydroxyl groups (Kulling, Honig, Simat, & Metzler, 2000). The phenolic hydroxyl group acts as an oxygen donor to react with free radicals in order to produce corresponding ions or molecules, extinguishing radicals and terminating the chain reaction of free radicals (Zuo et al., 2011). Thus, both genistein and daidzein are active substances exhibiting improved antioxidant activity. Daidzin and genistin could also play the role of antioxidants by inducing antioxidant enzymes to a certain extent (Chiang, Wang, & Chang, 2016).

In the current study, the total amount, activity, and antioxidative effect of soybean isoflavones in vitro were evaluated using LP‐CQPC01‐fermented soybean milk (LP‐CQPC01‐FSM). Meanwhile, animal experiments and molecular biological experiments were used to observe the inhibitory effect of LP‐CQPC01‐FSM on constipation in mice. Comparison with the commercial strain *L. bulgaricus*‐fermented soybean milk (LB‐FSM) as a control was conducted to verify the effects of LP‐CQPC01‐FSM and determine the mechanism underlying the inhibitory action on constipation through its antioxidative effects.

## 2 MATERIALS AND METHODS

### 2.1 Fermentation strain

The strain used for soybean milk fermentation was isolated and identified from Sichuan Paocai (pickled cabbage) in Sichuan, China, by our research team. Referred to as *Lactobacillus plantarum* CQPC01 (LP‐CQPC01), this strain is preserved in China General Microbiological Culture Collection Center (CGMCC) (Beijing, China) with the preservation number CGMCC No. 14490. *L. bulgaricus* (LB) is used as the control strain for soybean milk fermentation, preserved in China Center for Type Culture Collection (CCTCC) (Wuhan, Hubei, China) with the stain number CCTCC No. AB 200048.

### 2.2 Soybean milk fermentation

LP‐CQPC01 and LB were activated twice, and the strains were preserved. Soybeans (1 kg) were soaked in 3 times of water for 12 hr (Table 1). The proportion of soaked soybean to water was 1:3. The soaked soybean was beaten and filtered. LP‐CQPC01 and LB were finally inoculated in soybean milk according to the concentration 10^5^ CFU/ml, and the soybean milk was fermented at 37°C for 12 hr. The total lactic acid bacterial count was determined using the agar plate counting method (Jett, Hatter, Huycke, & Gilmore, 1997).

**Table 1:** Fermentation conditions of soybean milk

Groups	Soaking water (multiple of soybean quality)	Soaking time (hr)	Inoculation concentration of bacteria (10^5^ CFU/ml)	Fermentation temperature (°C)	Fermentation time (hr)
U‐FSM	3.0	12.0	/	37	12.0
LB‐FSM	3.0	12.0	1.0 LB	37	12.0
LP‐CQPC01‐FSM	3.0	12.0	1.0 LP‐CQPC01	37	12.0

Note

LB‐FSM: *Lactobacillus bulgaricus‐*fermented soybean milk; LP‐CQPC01‐FSM: *Lactobacillus plantarum* CQPC01‐fermented soybean milk; U‐FSM: unfermented soybean milk.

### 2.3 In vitro experiment of soybean milk

#### 2.3.1 High‐performance liquid chromatography assay

Standard daidzin, glycitin, genistin, daidzein, glycitein, and genistein (Yuanye Biotech Co., Ltd.) were prepared as standard solutions. The test was conducted under the following chromatographic conditions (Skyray Instrument LC‐CT310 HPLC workstation, Skyray Instrument): chromatographic column, Pntulips QS C18 chromatographic column (4.6 mm × 250 mm, 5 μm); mobile phase, acetonitrile–0.1% phosphoric acid; detection wavelength, 254 nm; column temperature, 40°C; flow rate, 1 ml/min; and sampling amount, 10 μl. The six main chromatographic peaks (daidzin, glycitin, genistin, daidzein, glycitein, and genistein) were obtained in 16.899, 18.298, 26.582, 39.483, 41.325, and 48.525 min, and the elution gradient is presented in Table 2. The fermented 2 ml soybean milk was dissolved in an 80% methanol solution and then transferred into a 50‐ml volumetric flask, adding 80% methanol to near calibration ultrasonically at 50°C for 1 hr. The ultrasonic sample was fixed with 80% methanol to 50 ml and shaken well. The sample was poured into a 10‐ml centrifuge tube and then centrifuged for 15 min (14,400 × *g*). The supernatant was filtered using a 0.45‐μm filter membrane, and the filtrate was collected for backup. Samples at 5 μl were detected by liquid chromatography. The isoflavone content of the soybean milk was calculated using the peak area (*Y*) and sample volume (*X*) by linear regression (Table 3).

**Table 2:** Ratio gradient elution of mobile phase

Time (min)	Mobile phase A (%)	Mobile phase B (%)
0	13	87
0–7	13 → 17	87 → 83
7–23	17 → 21	83 → 79
23–30	21 → 27	79 → 73
30–40	27 → 29	73 → 71
40–50	29 → 70	71 → 30

Note

Mobile phase A: acetonitrile; mobile phase B: 0.1% phosphoric acid.

**Table 3:** Standard curves of six kinds of soybean isoflavones

Standard	Regression equation	R^2^
Daidzin	*Y* = 498,451*X *– 11,455	0.9925
Glycitin	*Y* = 441,374*X *– 9,367.1	0.9893
Genistin	*Y* = 523,823X – 12,158	0.9896
Daidzein	*Y* = 785,168X – 19,192	0.9916
Glycitein	*Y* = 228,932*X *– 5,739.8	0.9918
Genistein	*Y* = 918,414X – 10,251	0.9894

#### 2.3.2 In vitro determination of antioxidative effect by DPPH assay

After the soybean milk was diluted five times (U‐FSM, LB‐FSM, and LP‐CQPC01‐FSM), A_1_ (3.9 ml DPPH solution and 100 µl soybean milk), A_2_ (3.9 ml anhydrous ethanol and 100 µl soybean milk), and A_3_ (3.9 ml DPPH solution and 100 µl 80% methanol solution) were reacted, and 200 µl reaction solution was removed and added to 96‐well plate. Then, the absorbance was measured at 517 nm and the antioxidative effect was calculated (Mohamed Mahzir et al., 2018). The formula of antioxidant activity determination by DPPH was as follows: DPPH clearance rate (%) = (OD517 of A3 − (OD517 of A1 − OD517 of A2))/OD517 of A3 × 100.

#### 2.3.3 In vitro determination of antioxidative effect by ABTS assay

After the soybean milk was diluted five times (U‐FSM, LB‐FSM, and LP‐CQPC01‐FSM), A_1_ (5 ml ABTS reaction solution and 200 µl soybean milk), A_2_ (5 ml anhydrous ethanol and 200 µl soybean milk), and A_3_ (5 ml ABTS solution and 200 µl 80% methanol solution) were reacted, and 200 µl reaction solution was removed and added to 96‐well plate. Then, the absorbance was measured at 734 nm and the antioxidative effect was calculated (Venturi et al., 2017). The formula of antioxidant activity determination by ABTS was as follows: ABTS clearance rate (%) = (OD734 of A3 − (OD734 of A1 − OD734 of A2))/OD734 of A3 × 100.

#### 2.3.4 In vitro determination of antioxidative effect by hydroxyl radical assay

After the soybean milk was diluted five times (U‐FSM, LB‐FSM, and LP‐CQPC01‐FSM), A_1_ (300 µl 80% methanol solution and 2.0 ml FeSO_4_ and 1.0 ml salicylic acid ethanol solution and 1.0 ml H_2_O_2_), A_2_ (300 µl soybean milk and 2.0 ml FeSO_4_ and 1.0 ml salicylic acid ethanol solution and 1.0 ml H_2_O_2_), and A_3_ (300 µl soybean milk and 2.0 ml FeSO_4_ and 1.0 ml salicylic acid ethanol solution and 1.0 ml 80% methanol solution) were reacted, and 200 µl reaction solution was removed and added to 96‐well plate. Then, the absorbance was measured at 510 nm and the antioxidative effect was calculated (Li, Sun, Gu, & Guo, 2018). The formula of antioxidant activity determination by hydroxyl radical assay was as follows: OH clearance rate (%) = (OD510 of A3 – (OD510 of A1 – OD510 of A2))/OD510 of A3 × 100.

### 2.4 In vivo experiment of soybean milk

#### 2.4.1 Animal experiment

Kunming mice (100, 7 weeks old, female) were purchased from Chongqing Medical University (Chongqing, China). This study was approved by the Animal Ethics Committee of Chongqing Medical University (SYXK (Yu) 2017–0001). After 1 week of adaptive feeding, the mice were used in the experiment. The mice were divided into five groups: the normal group, control group, group treated with unfermented soybean milk or U‐FSM, group treated with LB‐fermented soybean milk or LB‐FSM, and group treated with LP‐CQPC01‐fermented soybean milk or LP‐CQPC01‐FSM. The duration of the experiment was 2 weeks. The U‐FSM, LB‐FSM, and LP‐CQPC01‐FSM groups received treatment in these 2 weeks, in addition to the normal intake of free diet and drinking water fed to the mice daily to a total of 2 ml (divided into four feedings via gavage) of the corresponding soybean milk. Two weeks later, except for the normal group, the groups of mice were fed with 0.2 ml 10% activated carbon solution (activated carbon was added to the solution containing 10% gum Arabic by the mass fraction of 10%) daily by gavage for 3 days. Simultaneously, the U‐FSM, LB‐FSM, and LP‐CQPC01‐FSM groups continued being fed the corresponding soybean milk by gavage. The weight, particle count, and water content of the mouse stool were measured at 9:00 a.m. On the last day of the experiment, after activated carbon water was administered by gavage, each group was divided into 10 mice to observe the first black stool defecation time of mice. The remaining 10 mice in each group were asphyxiated with CO_2_. The blood and small intestinal tissues of the mice were kept at −20°C. The length of the small intestine and the propelling length of the activated carbon (length of GI transit) were determined, and the propulsion rate was calculated using the formula = (length of GI transit/length of small intestine) × 100% (Qian, Song, Sun, et al., 2018).

#### 2.4.2 Determination of MTL, Gas, ET, AchE, SP, and VIP serum levels

The collected mice plasma was centrifuged at 2,490 × *g* for 15 min. The MTL, Gas, ET, SS, AChE, SP, and VIP serum levels in mice were measured using kits (Nanjing Jiancheng Bioengineering Institute; Qian, Song, Sun, et al., 2018).

#### 2.4.3 Determination of small intestine tissue MPO, NO, MDA, and GSH levels

The small intestine tissues of the collected mice were treated with homogenate (S10, Ningbo Scientz Biotechnology Co., Ltd.). The MPO, NO, MDA, and GSH levels in the small intestine tissue were measured using kits (Nanjing Jiancheng Bioengineering Institute; Qian, Song, Sun, et al., 2018).

#### 2.4.4 Small intestine tissue hematoxylin and eosin staining of sections

Small intestine tissues of mice were soaked in 10% formalin fixative for hematoxylin and eosin staining. The tissue morphology was observed using a microscope (BX43F; Olympus). Changes in the small intestine tissues were observed to evaluate the effects of the soybean milk (Qian, Song, Sun, et al., 2018).

#### 2.4.5 Quantitative PCR assay

The RNA of the small intestine tissues of mice was extracted using the extractor RNAzol (Thermo Fisher Scientific). Total RNA was digested using RNase‐free water at 37°C for 15 min. The RNA was then purified using the RNeasy kit (Thermo Fisher Scientific) at the concentration of 1 μg/μl. The RNA template was synthesized to cDNA at 37°C for 120 min, 99°C for 4 min, and 4°C for 3 min. The mRNA expression levels (Table 4) were measured by real‐time quantitative PCR (qPCR; StepOnePlus, Thermo Fisher Scientific) to conduct 40 cycles at 95°C for 3 min (predenaturation), 95°C for 10 s (denaturation), 57°C for 30 s (annealing), and 72°C for 15 s (extension). The relative mRNA expression levels were ultimately calculated using the 2^−ΔΔCr^ formula (Qian, Song, Sun, et al., 2018; Qian, Song, Yi, et al., 2018).

**Table 4:** Sequences of primers used in this study

Gene name	Sequence
Cu/Zn‐SOD	Forward: 5′‐GAAGAGAGGCATGTTGGAGA‐3′
	Reverse: 5′‐CCAATTACACCACGAGCCAA‐3′
Mn‐SOD	Forward: 5′‐TTCAATAAGGAGCAGGGAC3′
	Reverse: 5′‐CAGTGTAAGGCTGACGGTTT‐3′
CAT	Forward: 5′‐GGAGGCGGGAACCCAATAG‐3′
	Reverse: 5′‐GTGTGCCATCTCGTCAGTGAA‐3′
c‐Kit	Forward: 5′‐CATAGCCCAGGTAAAGCACAAT‐3′
	Reverse: 5′‐GAACACTCCAGAATCGTCAACTC‐3′
SCF	Forward: 5′‐TCAGGGACTACGCTGCGAAAG‐3′
	Reverse: 5′‐AAGAGCTGGCAGACCGACTCA‐3′
TRPV1	Forward: 5′‐CCGGCTTTTTGGGAAGGGT‐3′
	Reverse: 5′‐GAGACAGGTAGGTCCATCCAC‐3′
GDNF	Forward: 5′‐GGGGTATGGAGAAGTTGGCTAG‐3′
	Reverse: 5′‐CTATGAGAATGCTGCCGAAAA‐3′
NOS	Forward: 5′‐CAGCGAACGGACGGCAAGCA‐3′
	Reverse: 5′‐TGACACGACCAGCGGCAGGAT‐3′
GAPDH	Forward: 5′‐TGCACCACCAACTGCTTAG‐3′
	Reverse: 5′‐GATGCAGGGATGATGTTC‐3′

#### 2.4.6 Western blot assay

The protein in the small intestine tissues in mice was extracted using the kit (Thermo Fisher Scientific), and the protein concentration was adjusted to 30 µg/ml. Sodium dodecyl phosphate–polyacrylamide gel electrophoresis was conducted using a 10% separation gel and a 5% stacking gel. The protein that was extracted from the small intestine tissues was isolated for 2 hr by using a 5% nonfat milk sealing liquid. The protein was then combined at 25°C for 2 hr using the primary antibodies of Cu/Zn‐SOD (MA1‐105, 1:1,000 dilution, Thermo Fisher Scientific), Mn‐SOD (LF‐MA0030, 1:1,000 dilution, Thermo Fisher Scientific), CAT (702732, 1:500 dilution, Thermo Fisher Scientific), c‐Kit (14–1172‐82, 1:1,000 dilution, Thermo Fisher Scientific), SCF (PA5‐20746, 1:1,000 dilution, Thermo Fisher Scientific), TRPV1 (PA5‐77317, 1:200 dilution, Thermo Fisher Scientific), GDNF (PA5‐77537, 1:1,000 dilution, Thermo Fisher Scientific), NOS (sc‐49058, 1:1,000 dilution, Santa Cruz Biotechnology, Inc.), and β‐actin (MA1‐140, 1:5,000 dilution, Thermo Fisher Scientific). After treatment with the primary antibody, the sample membrane was soaked in the secondary antibody (A32723, 1:5,000 dilution, Thermo Fisher Scientific) solution at 25°C for 1 hr. After washing the membrane three times using TBST, the GIS gel image was used to photograph the system (iBright™ FL1000 Imaging System, Thermo Fisher Scientific; Qian, Song, Sun, et al., 2018; Qian, Song, Yi, et al., 2018).

### 2.5 Statistical analysis

The normality of distribution of experimental data was analyzed by Kolmogorov–Smirnov test, and value in normal distribution was analyzed using the mean ± standard deviation. Significant differences (*p* < 0.05) in the experimental data for the different groups were analyzed by Duncan's multiple comparison test using SPSS ver. 12.0 (IBM Corporation).

## 3 RESULTS AND DISCUSSION

### 3.1 Soybean isoflavones in soybean milk

**Figure 1:**
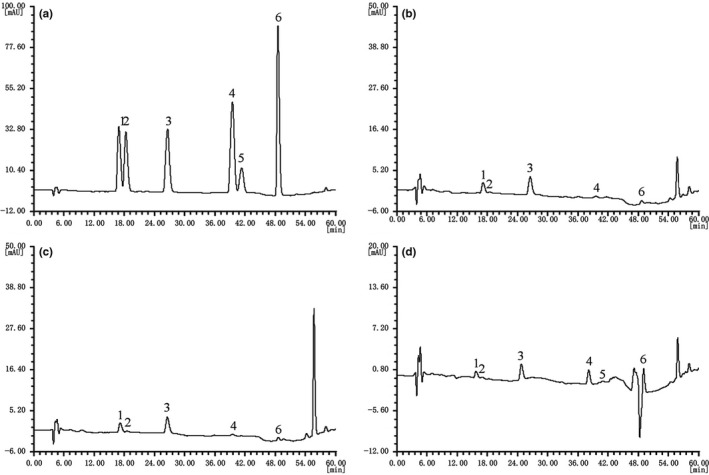
High‐performance liquid chromatography chromatography for soybean isoflavone in fermented soybean milk. (a): standard; (b): unfermented soybean milk; (c): *Lactobacillus bulgaricus‐*fermented soybean milk; (d): *Lactobacillus plantarum* CQPC01‐fermented soybean milk; 1: daidzin; 2: glycitin; 3: genistin; 4: daidzein; 5: glycitein; 6: genistein

After detection by high‐performance liquid chromatography (HPLC), unfermented soybean milk (U‐FSM) and *L. bulgaricus*‐fermented soybean milk (LB‐FSM) were found to contain daidzin, glycitin, genistin, daidzein, and genistein; meanwhile, *L. plantarum* CQPC01‐fermented soybean milk (LP‐CQPC01‐FSM) was found to contain more than five kinds of soybean isoflavones and glycitein (Figure 1). After calculation using the regression equation, LP‐CQPC01‐FSM was found to contain more free glycosides, compared with U‐FSM and LB‐FSM; however, the combined glycoside content of LP‐CQPC01‐FSM was less than that of U‐FSM and LB‐FSM (Table 5). The total soybean isoflavone content of LP‐CQPC01‐FSM was also more than that of U‐FSM and LB‐FSM. On the basis of these results, LP‐CQPC01 could produce more active soybean isoflavones compared with LB by fermentation.

**Table 5:** Soybean isoflavone contents in the fermented soybean milk (μg/ml)

Sample	Daidzin	Glycitin	Genistin	Combined glycosides	Daidzein	Glycitein	Genistein	Free glycosides
U‐FSM	4.69 ± 0.01^a^	2.41 ± 0.01^a^	6.57 ± 0.01^a^	13.67 ± 0.01^a^	2.63 ± 0.01^b^	0.00 ± 0.00^b^	1.38 ± 0.01^b^	4.01 ± 0.01^c^
LB‐FSM	4.36 ± 0.02^b^	2.40 ± 0.01^a^	6.13 ± 0.01^b^	12.89 ± 0.02^b^	2.65 ± 0.01^b^	0.00 ± 0.00^b^	1.40 ± 0.01^b^	4.05 ± 0.01^b^
LP‐CQPC01‐FSM	3.04 ± 0.01^c^	2.30 ± 0.01^b^	4.12 ± 0.02^c^	9.46 ± 0.01^c^	3.45 ± 0.01^a^	2.70 ± 0.01^a^	4.30 ± 0.01^a^	10.45 ± 0.02^a^

Note

^a–c^Mean values with different letters in the same column are significantly different (*p* < 0.05) according to Duncan's multiple range test. LB‐FSM: *Lactobacillus bulgaricus*‐fermented soybean milk; LP‐CQPC01‐FSM: *Lactobacillus plantarum* CQPC01‐fermented soybean milk; U‐FSM: unfermented soybean milk.

Soybean isoflavones could promote the differentiation of intestinal gland cells and the development of small intestinal epithelial cells, improve the intestinal microbial environment, reduce the stimulation of harmful antigen to the intestine, and keep the small intestine healthy (Chen, Hao, & Xiao, 2014). Soybean isoflavones could also increase the beneficial bacteria in the intestinal tract (Clavel et al., 2005) and promote the proliferation of colonic epithelial cells (Adams et al., 2005). The beneficial effects of soybean isoflavones on intestinal tract may play a role in inhibiting constipation. In clinical research, the intake of soy isoflavones was positively related to the effect on the intestinal tract: Adults daily intake of 83 mg soybean isoflavones could protect the intestines, which is equivalent to 1.4 mg per kilogram of body weight (Adams et al., 2005). In this study, the intake of soybean isoflavones in mice was about 1 mg per kilogram of body weight, which was equivalent to 1/10 of the clinical intake of human body. It could be seen that soybean isoflavones could produce better results in the case of low intake of soybean isoflavones, and the intake of certain amount of soybean milk, in accordance with the drinking habit, might help the human body to inhibit constipation.

Genistein, daidzein, daidzin, and genistin are soybean isoflavones, and they have antioxidant activities (Röhrdanz, Ohler, Tranthi, & Kahl, 2002). In the current study, the genistein content of LP‐CQPC01‐FSM was three times that of LB‐FSM and U‐FSM. LP‐CQPC01‐FSM also contained daidzein, which were not detected in LB‐FSM and U‐FSM. These two soybean isoflavones in LP‐CQPC01‐FSM were slightly lower than those in LB‐FSM and U‐FSM. The study also showed that daidzein played an antioxidant role via the antioxidant enzyme system and could protect tissues from damage (Song, Yan, & Cai, 2006). Daidzein was slightly higher in LP‐CQPC01‐FSM than in either LB‐FSM or U‐FSM, and could contribute to the antioxidant activity of LP‐CQPC01‐FSM. Soybean isoflavones also exert an apparent antioxidative effect on the entire animal, and their extracts clearly inhibit the increase in the peroxide level and the decrease in antioxidant enzyme activity in mice induced by doxorubicin (Cohen et al., 2007). A study also showed that soybean isoflavones could promote the proliferation of probiotics in the intestine, thereby inhibiting constipation (Setchell et al., 2002). In addition, combined glycosides released the free form of isoflavone glycoside under the action of intestinal bacteria and were absorbed by the body at the upper end of the small intestine, whereas free glycosides could be absorbed directly at the upper end of the small intestine (Wang et al., 2017). The free glycoside content in LP‐CQPC01‐FSM was 2.5 times higher than that in LB‐FSM and U‐FSM, which could be more easily absorbed and used to perform certain functions in the body. The inhibitory effect of soy milk on constipation may mainly be attributed to the difference in soybean isoflavones.

### 3.2 In vitro antioxidative effect of soybean milk

**Figure 2:**
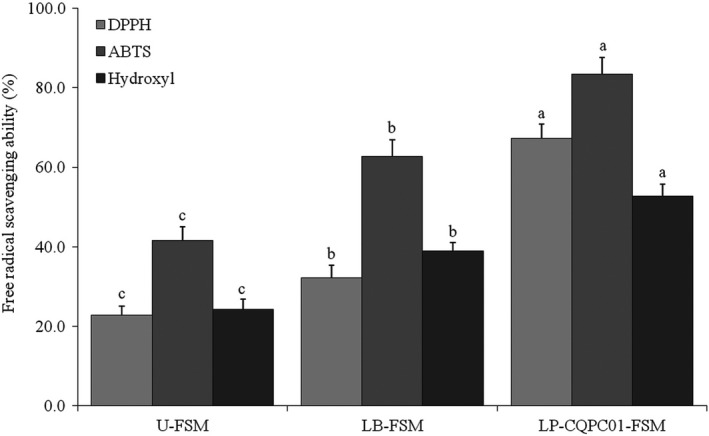
DPPH, ABTS, and hydroxyl radical scavenging activities of soybean milk. ^a–e^Mean values with different letters in the same bars are significantly different (*p* < 0.05) according to Duncan's multiple range test. LB‐FSM: *Lactobacillus bulgaricus*‐fermented soybean milk; LP‐CQPC01‐FSM: *Lactobacillus plantarum* CQPC01‐fermented soybean milk; U‐FSM: unfermented soybean milk

Through in vitro experiments, LP‐CQPC01‐FSM was determined to exhibit the best DPPH, ABTS, and hydroxyl radical scavenging abilities, followed by LB‐FSM and then U‐FSM (Figure 2). The fermented soybean milk exerted better in vitro antioxidative effects compared with U‐FSM; compared with LB, LP‐CQPC01 could improve the in vitro antioxidative effects of soybean milk by fermentation.

### 3.3 Determination of the total number of bacteria

After fermentation for 12 hr, the total number of bacteria in LP‐CQPC01‐FSM increases to 6.2 × 10^9^ CFU/ml, and the total number of bacteria in LB‐FSM also rises to 6.0 × 10^9^ CFU/ml. No significant difference (*p* > 0.05) in the proliferation rate was determined between these two kinds of lactic acid bacteria in soybean milk.

### 3.4 Body weight of mice

**Figure 3:**
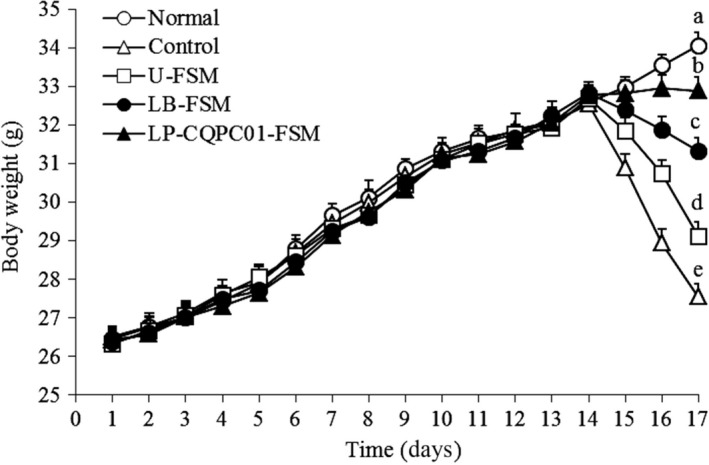
Body weight of mice during the experiment. ^a–e^Mean values with different letters in the same bars are significantly different (*p* < 0.05) according to Duncan's multiple range test. LB‐FSM: *Lactobacillus bulgaricus*‐fermented soybean milk; LP‐CQPC01‐FSM: *Lactobacillus plantarum* CQPC01‐fermented soybean milk; U‐FSM: unfermented soybean milk

As shown in Figure 3, from the first to the fourteenth day, the weight of mice in each group increased normally, but there was no significant difference (*p* > 0.05). After constipation induced by activated carbon solution, the weight of mice began to decrease. The body weight of mice was reduced by intragastric administration of soybean milk, and LP‐CQPC01‐FSM showed the better effect than LB‐FSM and U‐FSM.

Constipation affects appetite and can lead to weight loss. Therefore, weight loss is also an important indicator of constipation (Mohamed Mahzir et al., 2018). Soybean milk could inhibit the body weight loss, and LP‐CQPC01‐FSM had the best effect: It could inhibit the experimental constipation in mice. Probiotics play an important role in the intestinal tract, which is directly related to constipation (Ding et al., 2016; Suo et al., 2014). LP‐CQPC01‐FSM and LB‐FSM have almost the same total number of bacteria. The inhibitory effect of soy milk on constipation may mainly be attributed to the difference in soybean isoflavones.

### 3.5 Stool status of mice

As shown in Table 6, the weight, particle count, and water content of mouse stool in different groups have no significant difference (*p* > 0.05) between each group before constipation is induced (1–14 days). After constipation is induced (15–17 days), the weight, particle count, and water content of mouse stool in the normal group are greater than those of the mice in other groups. However, these indexes of mice are lowest in the control group. The LP‐CQPC01‐FSM group has higher values for weight, particle count, and water content of stool mouse stool than those of the mice in the LB‐FSM and U‐FSM groups. LP‐CQPC01‐FSM can most reduce the influence of constipation on defecation, followed by LB‐FSM and then U‐FSM.

**Table 6:** Stool status of mice treated with soybean milk during the experiment

Groups	Normal	Control	U‐FSM	LB‐FSM	LP‐CQPC01‐FSM
1–14 days (lactic acid bacteria administration period but not induction of constipation)					
Stool weight (g)	0.96 ± 0.05^a^	0.95 ± 0.05^a^	0.95 ± 0.04^a^	0.95 ± 0.05^a^	0.96 ± 0.04^a^
Particle count of stool	41 ± 3^a^	42 ± 4^a^	42 ± 4^a^	41 ± 2^a^	41 ± 3^a^
Water content of stool (%)	52 ± 5^a^	53 ± 4^a^	53 ± 3^a^	55 ± 5^a^	56 ± 4^a^
15–17 days (lactic acid bacteria administration period, induction of constipation)					
Stool weight (g)	0.97 ± 0.03^a^	0.44 ± 0.07^e^	0.62 ± 0.06^d^	0.73 ± 0.04^c^	0.81 ± 0.04^b^
Particle count of stool	43 ± 4^a^	20 ± 3^d^	28 ± 5^c^	33 ± 3^bc^	38 ± 3^b^
Water content of stool (%)	53 ± 4^a^	18 ± 4^d^	29 ± 4^c^	35 ± 4^bc^	46 ± 3^b^

Note

Values presented are the mean ± standard deviation (*N* = 10/group). ^a–d^Mean values with different letters in the same row are significantly different (*p* < 0.05) according to Duncan's multiple range test. LB‐FSM group: mice treated with *Lactobacillus bulgaricus‐*fermented soybean milk; LP‐CQPC01‐FSM group: mice treated with *Lactobacillus plantarum* CQPC01‐fermented soybean milk; U‐FSM group: mice treated with unfermented soybean milk.

Constipation is characterized by the reduced frequency of defecation, difficulty of defecation, and dryness in stool (Gibson & Roberfroid, 2004). The mice with activated carbon‐induced constipation could cause the aforementioned symptoms. LP‐CQPC01‐FSM could increase the weight, particle count, and water content of stool in constipated mice, and these increases were considerably higher than those of other soybean milk.

### 3.6 First black stool defecation time of mice

**Figure 4:**
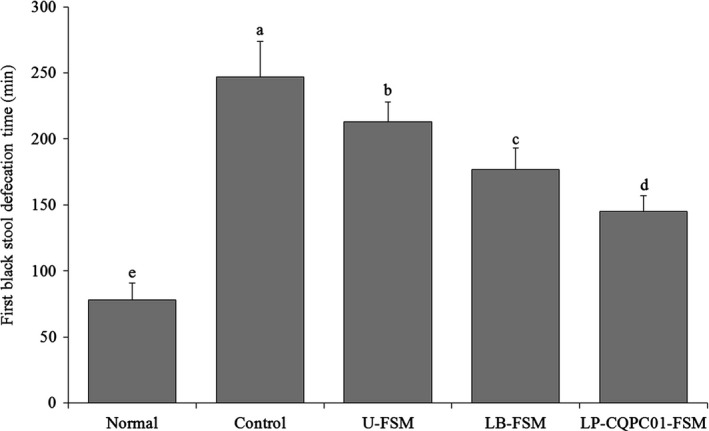
First black stool defecation time of the mice.^a–e^ Mean values with different letters in the same bars are significantly different (*p* < 0.05) according to Duncan's multiple range test. LB‐FSM: *Lactobacillus bulgaricus‐*fermented soybean milk; LP‐CQPC01‐FSM: *Lactobacillus plantarum* CQPC01‐fermented soybean milk; U‐FSM: unfermented soybean milk

As shown in Figure 4, the first black stool defecation time of mice in the normal group is the shortest at 78 min, whereas that in the control group is the longest at 247 min. The first black stool defecation time of mice in the following groups is as follows: U‐FSM, 145 min; LB‐FSM, 177 min; and LP‐CQPC01‐FSM, 213 min.

Constipation decreases the frequency of intestinal peristalsis and increases the length of time feces remain in the intestines. This effect prompts harmful bacteria to reproduce continuously with feces, threatening intestinal health and aggravating constipation (Feighner et al., 1999). After activated carbon was used to induce constipation, gastrointestinal transit could be used as an indicator for evaluating the activity and constipation of the small intestine (Setchell et al., 2002). The gastrointestinal transit of LP‐CQPC01‐FSM was longer than that of other soybean milk. LP‐CQPC01‐FSM has been proved to exert a good inhibitory effect on constipation. Constipation slows bowel movement, increases the transit time and dimension of the intestine, and lengthens the time of the first black stool. The sooner the first black stool is expelled, the greater is the tendency toward normal intestinal movement (Setchell et al., 2002). In the present study, compared with the constipation control group, LP‐CQPC01‐FSM could significantly reduce the time of first black stool discharge and helped relieve constipation.

### 3.7 Gastrointestinal transit abilities of mice

As shown in Table 7, no significant difference (*p* > 0.05) in the length of small intestine is found between groups. The mice in the normal group exhibit the highest GI transit ability at 91.5%, followed by the mice in the LP‐CQPC01‐FSM (84.0%), LB‐FSM (70.8%), and U‐FSM (60.0%) groups. These results suggest that LP‐CQPC01‐FSM exhibits the highest increases in GI transit ability, followed by LB‐FSM and then U‐FSM after constipation is induced.

**Table 7:** Gastrointestinal (GI) transit in mice with activated carbon‐induced constipation

Groups	Length of small intestine (cm)	Length of GI transit (cm)	Activated carbon propulsive rate (%)
Normal	42.3 ± 2.1^a^	38.7 ± 2.0^a^	91.5 ± 1.2^a^
Control	42.1 ± 1.8^a^	8.2 ± 2.1^e^	19.5 ± 2.3^e^
U‐FSM	43.0 ± 2.2^a^	25.8 ± 2.5^d^	60.0 ± 2.1^d^
LB‐FSM	42.8 ± 2.4^a^	30.3 ± 2.2^c^	70.8 ± 2.1^c^
LP‐CQPC01‐FSM	41.9 ± 2.7^a^	35.2 ± 1.3^b^	84.0 ± 1.8^b^

Note

Values presented are the mean ± standard deviation (*N* = 10/group). ^a–e^Mean values with different letters in the same column are significantly different (*p* < 0.05) according to Duncan's multiple range test. LB‐FSM group: mice treated with *Lactobacillus bulgaricus‐*fermented soybean milk; LP‐CQPC01‐FSM: mice treated with *Lactobacillus plantarum* CQPC01‐fermented soybean milk; U‐FSM group: mice treated with unfermented soybean milk.

### 3.8 Serum MTL, Gas, ET, SS, AchE, SP, and VIP levels in mice

The MTL, Gas, ET, AchE, SP, and VIP serum levels of mice in the normal group are the highest (Table 8), and the serum SS level was the lowest. Constipation reduces the MTL, Gas, ET, AchE, SP, and VIP levels but increases the SS level. LP‐CQPC01‐FSM can improve the MTL, Gas, ET, AchE, SP, and VIP levels and reduce the SS level after constipation is induced in the mice. These effects are the highest, followed by those of LB‐FSM and U‐FSM.

**Table 8:** MTL, GAS, ET, SS, AchE, SP, and VIP serum levels in mice with activated carbon‐induced constipation

Levels (pg/mL)	Normal	Control	U‐FSM	LB‐FSM	LP‐CQPC01‐FSM
MTL	217.6 ± 19.8^a^	77.8 ± 6.5^e^	108.7 ± 11.9^d^	141.6 ± 16.2^c^	171.2 ± 12.3^b^
Gas	115.3 ± 10.3^a^	33.6 ± 5.1^e^	69.8 ± 6.3^d^	79.9 ± 6.1^c^	93.8 ± 7.3^b^
ET	27.7 ± 2.6^a^	5.3 ± 0.5^e^	9.7 ± 0.8^d^	17.4 ± 1.8^c^	23.6 ± 1.4^b^
SS	22.0 ± 3.6^e^	79.3 ± 4.1^a^	58.7 ± 3.3^b^	41.6 ± 3.8^c^	32.6 ± 2.2^d^
AchE	42.3 ± 3.3^a^	9.7 ± 1.2^e^	19.3 ± 1.8^d^	29.7 ± 2.2^c^	36.1 ± 1.5^b^
SP	88.5 ± 4.2^a^	25.7 ± 2.0^e^	45.3 ± 3.9	59.9 ± 4.3^c^	71.8 ± 3.6^b^
VIP	82.6 ± 3.7^a^	16.3 ± 1.9^e^	33.2 ± 2.9^d^	48.7 ± 3.3^c^	67.9 ± 3.0^b^

Note

Values presented are the mean ± standard deviation (*N* = 10/group). ^a–e^Mean values with different letters in the same column are significantly different (*p* < 0.05) according to Duncan's multiple range test. AchE: acetylcholinesterase; ET, endothelin; Gas: gastrin; LB‐FSM group: mice treated with *Lactobacillus bulgaricus*‐fermented soybean; LP‐CQPC01‐FSM group: mice treated with *Lactobacillus plantarum* CQPC01‐fermented soybean milk; MTL: motilin; SP: substance P; SS: somatostatin; U‐FSM group: mice treated with unfermented soybean milk; VIP: vasoactive intestinal peptide.

Motilin can stimulate the production of pepsin and promote intestinal motility (Preston, Adrian, Christofides, Lennard‐Jones, & Bloom, 1985). Gas can promote gastrointestinal (GI) secretion and gastrointestinal motility, promote pyloric relaxation, and relieve constipation (Soudah, Hasler, & Owyang, 1991). ET helps stabilize blood vessel tension and maintain the basic cardiovascular system (Furchgott & Zawadzki, 1980; Soudah et al., 1991). SS has been used to stimulate intestinal motility, which can help relieve constipation (Preston et al., 1985). AChE regulates muscle contraction and mucus secretion, which can relax muscles and promote fecal excretion (Tzavella et al., 1996). In addition, SP contributes to intestinal peristalsis (Milner, Crowe, Kamm, Lennard‐Jones, & Burnstock, 1990). Maintaining normal VIP content in the intestinal wall is an important means of stabilizing intestinal function (Augustin & Lutz, 1991). The results of this study also showed that LP‐CQPC01‐FSM could normalize these serum levels and relieve constipation, and it exerted greater effects, compared with LB‐FSM and U‐FSM.

### 3.9 Small intestine tissue MPO, NO, MDA, and GSH levels in mice

The MPO, NO, MDA levels in the small intestine tissue of constipated mice (control group) are the highest, whereas the GSH level is the lowest (Table 9). Soybean milk can reduce the MPO, NO, and MDA levels as well as increase the GSH level in the small intestine tissue of constipated mice. LP‐CQPC01‐FSM exhibited the most effects, followed by LB‐FSM and then U‐FSM.

**Table 9:** MPO, NO, MDA, and GSH levels in the small intestine tissue of mice with activated carbon‐induced constipation

Group	MPO (mU/mg)	NO (μmol/gprot)	MDA (nmol/mg)	GSH (μmol/mg)
Normal	4.2 ± 0.3^e^	0.5 ± 0.1^e^	0.4 ± 0.1^e^	9.3 ± 0. 4^a^
Control	19.7 ± 0.5^a^	2.6 ± 0.3^a^	1.6 ± 0.2^a^	4.2 ± 0.3^e^
U‐FSM	12.3 ± 0.3^b^	1.8 ± 0.3^b^	1.1 ± 0.2^b^	6.5 ± 0.3^d^
LB‐FSM	9.7 ± 0.4^c^	1.1 ± 0.2^c^	0.9 ± 0.1^c^	7.6 ± 0.2^c^
LP‐CQPC01‐FSM	6.0 ± 0.3^d^	0.8 ± 0.1^d^	0.7 ± 0.2^d^	8.5 ± 0.3^b^

Note

Values presented are the mean ± standard deviation (*N* = 10/group). ^a–e^Mean values with different letters in the same column are significantly different (*p* < 0.05) according to Duncan's multiple range test. GSH: glutathione; LB‐FSM group: mice treated with *Lactobacillus bulgaricus*‐fermented soybean milk; LP‐CQPC01‐FSM: mice treated with *Lactobacillus plantarum* CQPC01‐fermented soybean milk; MDA: malondialdehyde; MPO: myeloperoxidase; NO: nitric oxide; U‐FSM group: mice treated with unfermented soybean milk.

Activated carbon can damage the intestines to a certain degree and cause intestinal mucosal damage (Chen et al., 2018). Increased MPO activity in the intestine indicates a decrease in neutrophil aggregation and the occurrence of long‐tissue damage in tissues (Pedoto et al., 2001). During injury of the small intestine tissue, the formation of NO by iNOS can aggravate damage to the colon tissue; however, as the NO content increases, the activity of MPO increases and the damage is aggravated (Osman, Adawi, Ahrné, Jeppsson, & Molin, 2008). Intestinal damage can also lead to a large number of active oxygen ROS (reactive oxygen species) and active nitrogen RNS (reactive nitrogen species) and then to oxidative stress toxicity, causing damage in the intestinal tissue (Fiocchi, 2004). The formation of ROS after tissue damage destroys the oxidation/antioxidant balance of the body and reduces the GSH content in the intestinal tissue. A large amount of lipid peroxidation exacerbates the generation of the final product of lipid peroxidation (MDA; Ambrosone et al., 2005). As observed through these indicators, LP‐CQPC01‐FSM might relieve constipation by reducing intestinal tissue damage.

### 3.10 Morphological observation of small intestine tissue

**Figure 5:**
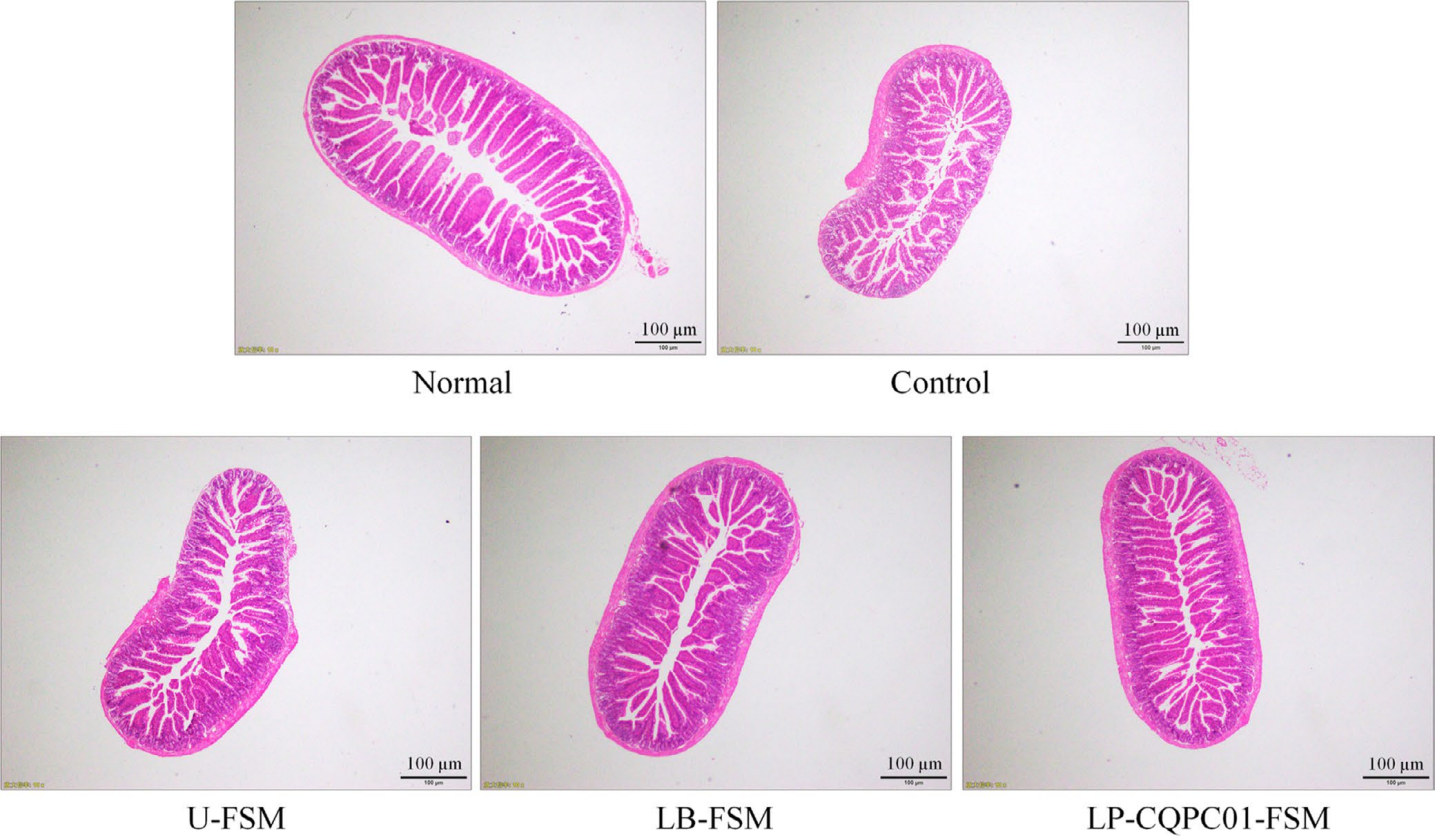
Morphological observation of small intestine tissue in mice with activated carbon‐induced constipation. LB‐FSM group: mice treated with *Lactobacillus bulgaricus‐*fermented soybean milk; LP‐CQPC01‐FSM group: mice treated with *Lactobacillus plantarum* CQPC01‐fermented soybean milk; U‐FSM group: mice treated with unfermented soybean milk

As shown in Figure 5, small intestine villi in the normal mice are arranged; the cells are also evenly distributed. After treatment with activated carbon (the control group), the wall of the small intestine becomes thinner, and the villus of the small intestine is broken. Meanwhile, the tissue cells in the small intestine also appear damaged. LP‐CQPC01‐FSM, LB‐FSM, and U‐FSM can reduce the damage in the small intestine after treatment with activated carbon. LP‐CQPC01‐FSM can restore most of the damage caused by activated carbon, followed by LB‐FSM and U‐FSM.

### 3.11 mRNA and protein expression levels of Cu/Zn‐SOD, Mn‐SOD, and CAT in small intestine tissue

**Figure 6:**
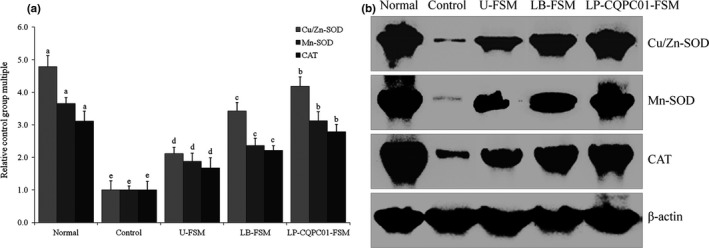
mRNA and protein expression levels of Cu/Zn‐SOD, Mn‐SOD, and CAT in the small intestine tissue of mice. Values presented are the mean ± standard deviation (*N* = 10/group). ^a–e^Mean values with different letters in the same bars are significantly different (*p* < 0.05) according to Duncan's multiple range test. LB‐FSM group: mice treated with *Lactobacillus bulgaricus‐*fermented soybean milk; LP‐CQPC01‐FSM group: mice treated with *Lactobacillus plantarum* CQPC01‐fermented soybean milk; U‐FSM group: mice treated with unfermented soybean milk

As shown in Figure 6, the mice in the normal group exhibit the highest expression levels of Cu/Zn‐SOD, Mn‐SOD, and CAT in the small intestine tissue, whereas the mice in the control group exhibit the lowest expression levels. After the mice are treated with soybean milk, the Cu/Zn‐SOD, Mn‐SOD, and CAT expression levels in the small intestine tissue of the constipated mice are increased; fermented soybean milk (LP‐CQPC01‐FSM and LB‐FSM) exerts stronger effects than U‐FSM, with the effects of LP‐CQPC01‐FSM being better than those of LB‐FSM.

Oxidative damage in the body can be defended in two ways: enzymatic and non‐enzymatic antioxidants. The regulation of SOD and CAT is the main mechanism of antioxidant enzymes (Morel, Hessler, & Chisolm, 1983). SOD catalyzes the dismutation of superoxide radicals, which can scavenge free radicals, and CAT and SOD play a synergistic role in enhancing the role of free radicals (Yao & Rarey, 1996). CAT, an important antioxidant enzyme in the body, can clear H_2_O_2_ in the body, thereby inhibiting oxidative stress (Gao, 2009). Mn‐SOD and Gu/Zn‐SOD are the isomers of SOD in the body. Mn‐SOD is a free radical scavenger on SOD, with Mn^4+ ^as the active center in the mitochondrion; Gu/Zn‐SOD is another free radical scavenger on SOD, which exists in the cytoplasm, with Cu^2+^ and Zn^2+^ as active centers (Saiki et al., 2007). Gu/Zn‐SOD can remove the O_2_^−^ toxic effect in the body and protect the visceral tissue (Bödör, Matolcsy, & Bernáth, 2010). Studies have shown that constipation also causes oxidative stress in the body to produce free radicals; Mn‐SOD and Gu/Zn‐SOD can inhibit the free radicals in the body and help prevent tissue damage (Kim et al., 2018; Wu, Yu, Sheng, & Lin, 2016). Patients with functional constipation often suffer from varying degrees of inflammation due to prolonged retention and irritation. Therefore, respiratory bursts during polymorphonuclear neutrophils and other phagocytic foreign bodies can produce large amounts of free radicals. With the aggravation of constipation, oxygen free radical reactions and lipid peroxidation are further initiated (Greer & Okeefe, 2011). The accumulation of large amounts of free radicals in the body also causes imbalance in the intestinal microenvironment and decreases in intestinal probiotics (Sanders, Koh, & Ward, 2006). Soybean milk not only exhibits the antioxidant properties of soybean isoflavones; the lactic acid bacteria in soybean milk can also enter the gut to improve the intestinal microecology. Specifically, LP‐CQPC01 exerts better effects against gastric juice and bile salt, compared with LB (unpublished data), and can play a more active role in the intestinal tract.

### 3.12 mRNA and protein expression levels of c‐Kit and SCF in small intestine tissue

**Figure 7:**
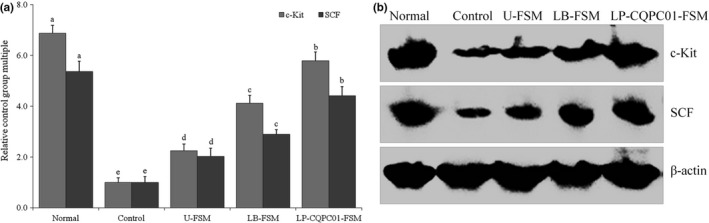
mRNA and protein expression levels of c‐Kit and CSF in the small intestine tissue of mice. **V** alues presented are the mean ± standard deviation (*N* = 10/group). ^a–e^Mean values with different letters in the same bars are significantly different (*p* < 0.05) according to Duncan's multiple range test. LB‐FSM group: mice treated with *Lactobacillus bulgaricus‐*fermented soybean milk; LP‐CQPC01‐FSM group: mice treated with *Lactobacillus plantarum* CQPC01‐fermented soybean milk; U‐FSM group: mice treated with unfermented soybean milk

As shown in Figure 7, the c‐Kit and SCF in the small intestine of the control group exhibit the lowest mRNA and protein expression levels. U‐FSM can increase these expression levels in mice after inducing constipation. Treatment with soybean milk after fermentation can further increase these expression levels. LP‐CQPC01‐FSM can increase these expression levels closest to that of the normal group, followed by LB‐FSM and U‐FSM.

Interstitial cells of Cajal (ICC) acts as the pacemaker that generates slow waves in the intestine and plays an important role in intestinal nerve signal transmission. ICC indeed affects the gastrointestinal function (Lyford et al., 2002). A study showed that the ICC density in the intestinal tract of patients with constipation was lower than that of patients in the normal state, which led to decreases in postsynaptic reaction between ICC and neurotransmitters, resulting in the loss of the action of spontaneous rhythmic slowed wave in ICC, irregular colon movement, and influence of the intestinal function (Farrugia, 2010). c‐Kit is a specific marker of ICC and is crucial to ICC proliferation (Brading & Mccloskey, 2005). SCF concentration is essential for ICC reproduction, without which ICC cannot grow. An animal experiment also showed that the ICC content and the expression level of c‐Kit in the intestinal tissue of constipated mice were reduced (Tong, Liu, Zhang, Zhang, & Lei, 2004). Lactic acid bacteria was also shown to effectively enhance the c‐Kit content and increase the ICC content in the intestinal tract of constipated mice, promote intestinal peristalsis, and relieve constipation (Geppetti & Trevisani, 2004). Soybean milk could not only regulate the expression levels of c‐Kit and SCF through its soybean isoflavones but could also promote intestinal peristalsis through the lactic acid bacteria in soybean milk, thereby alleviating constipation. The results showed that the effects of LP‐CQPC01‐FSM were markedly better than those of LB‐FSM.

### 3.13 mRNA and protein expression levels of TRPV1, GDNF, and NOS in small intestine tissue

**Figure 8:**
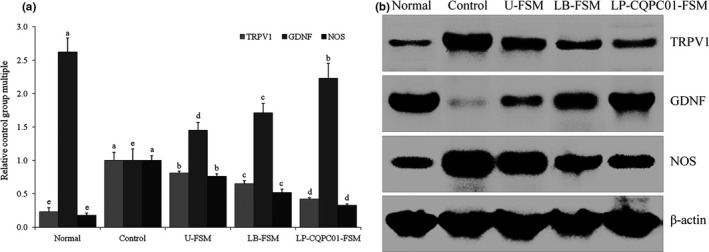
mRNA and protein expression levels of TRPV1, GDNF, and NOS in the small intestine tissue of mice. Values presented are the mean ± standard deviation (*N* = 10/group). ^a–e^Mean values with different letters in the same bars are significantly different (*p* < 0.05) according to Duncan's multiple range test. LB‐FSM group: mice treated with *Lactobacillus bulgaricus‐*fermented soybean milk; LP‐CQPC01‐FSM group: mice treated with *Lactobacillus plantarum* CQPC01‐fermented soybean milk; U‐FSM group: mice treated with unfermented soybean milk

As shown in Figure 8, the TRPV1 and NOS mRNA and protein expression levels of mice in the normal group are the lowest, although the GDNF mRNA and protein expression levels are the highest. After constipation is induced, the TRPV1, NOS mRNA, and protein expression levels are increased, and the GDNF mRNA and protein expression levels are decreased relative to those of the normal group. Soybean milk can inhibit changes in TRPV1, increase NOS expression, and reduce GNDF expression. LP‐CQPC01‐FSM exhibits the strongest inhibitory effects, followed by LB‐FSM and U‐FSM.

TRPV1 has been proved to be closely related to defecation. Activation of TRPV1 can trigger neurotransmitter release, resulting in intestinal motility disorder. Increased expression of TRPV1 is a significant phenomenon of intestinal injury. Intestinal injury caused by gastrointestinal disorders leads to increased TRPV1 expression in patients with constipation (Shah, Lyford, Gores, & Farrugia, 2004). GDNF can regulate the function of ganglion cells, helping repair damaged intestine and prevent constipation. Constipation is associated with the intestinal nervous system, which has NO as a major inhibitory neurotransmitter. NO relaxes the smooth muscles and weakens gastrointestinal movement. The increase in nitric oxide synthase (nitric oxide synthase, NOS)‐positive fibers increases the NO content, affecting intestinal function and constipation (Xiao & Tang, 2017). NOS participates in the regulation of gastrointestinal motility (Tomita, Igarashi, Fujisaki, & Tanjoh, 2007). An increase in NO can cause a more serious colon motility disorder. Reducing NO content by controlling NOS is a feasible method to control constipation (Xiao & Tang, 2017). Regulating the expression levels of TRPV1, GDNF, and NOS to relieve constipation is one of the mechanisms underlying the inhibitory role of soybean milk in constipation. Soybean milk participates in controlling constipation through its isoflavones and lactic acid bacteria. LP‐CQPC01‐fermented soybean milk exerted better effects, compared with the LB‐fermented soybean milk. The focus of this study was the effects of different strains on the contents of soybean isoflavones in fermented soybean milk, especially on the effects of constipation. Compared with previous studies, the effects of soybean isoflavones on the constipation in soybean milk were studied, but this study only carried out the mouse experiment, and we need further experiments on human body to get more accurate results.

## 4 CONCLUSIONS

A new *Lactobacillus* strain was found in Sichuan Paocai by our team, and it was referred to as LP‐CQPC01. In this study, the anticonstipation effects of LP‐CQPC01‐FSM were first researched. LP‐CQPC01‐FSM had the good anticonstipation effects in vivo, and its effects were better than common LB‐fermented soybean milk (LB‐FSM). LP‐CQPC01 could provide the soybean milk with more soybean isoflavones, particularly the active soybean isoflavones, relative to those of the common commercial strain of LB: The higher the active substance contents, the greater the inhibitory effect of the soybean milk on constipation. LP‐CQPC01 might be used as a new starter for high‐quality soybean‐fermented functional food. The mechanism of LP‐CQPC01 will also be strengthened in the future to accumulate theoretical foundation for making full use of this resource.

## CONFLICT OF INTEREST

The authors of this manuscript state that they do not have conflict of interest to declare.

## ETHICAL REVIEW

This study has not any potential sources of conflict of interest. This study was conducted in accordance with the Declaration of Helsinki, and the protocol was approved by the Ethics Committee of Chongqing Collaborative Innovation Center for Functional Food, China.
